# Discovery of the male of *Lobrathium rotundiceps* (Koch), and a new species of *Lobrathium* from Jiangxi, East China (Coleoptera, Staphylinidae, Paederinae)

**DOI:** 10.3897/zookeys.348.6299

**Published:** 2013-11-08

**Authors:** Wen-Rong Li, Li-Zhen Li

**Affiliations:** 1 Department of Biology, College of Life and Environmental Sciences, Shanghai Normal University, Shanghai, 200234, P. R. China

**Keywords:** *Lobrathium rotundiceps*, taxonomy, male, new species, China

## Abstract

The male of *Lobrathium rotundiceps* (Koch, 1939) from Zhejiang and *L. luoxiaoense* **sp. n.** from Jiangxi are described and illustrated.

## Introduction

*Lobrathium rotundiceps* was described by Koch (1939) as *Lathrobium rotundiceps* based on a single female collected from “Tienmuschan N.W. China”, but never recorded again since. Assing (2012) revised, redescribed, and illustrated the female holotype. The male sexual characters, however, which are of great significance for taxonomy of *Lobrathium* species, were unknown. Recently, additional specimens of *Lobrathium rotundiceps*, among them males, were collected in Zhejiang, or found in the collections of Shanghai Normal University.

In July 2013, two colleagues sifted two specimens of an undescribed *Lobrathium* in Jiangxi.

## Material and methods

The specimens treated in this study are deposited in the following public collections: The Insect Collection of Shanghai Normal University, Shanghai, P. R. China (SNUC); Naturhistorisches Museum Basel, Switzerland (NHMB).

The labels of type specimens are cited in their original spelling. A slash (/) is used to separate different labels.

All measurements are in millimeters. The following abbreviations are used: BL–length of the body from the anterior margin of the mandibles (in resting position) to the abdominal apex; HL–length of the head from the anterior margin of the frons to the posterior margin of the head; HW–maximum width of the head; PL–length of the pronotum along the midline; PW–maximum width of the pronotum; EL–length of the elytra from the anterior margin to the posterior elytral margin along suture; EW–maximum width of the elytra; AL–length of the aedeagus from the apex of the ventral process to the base of the aedeagal capsule.

## Taxonomy

### Lobrathium rotundiceps

(Koch, 1939)

http://species-id.net/wiki/Lobrathium_rotundiceps

Figs 1 , 2

Lathrobium rotundiceps Koch, 1939: 163.
Lobrathium rotundiceps (Koch): Assing 2012: 107 (redescription).

#### Type material studied.

Holotype ♀: “Tienmuschan, N.W. China Rtt. / Type / *Lathrobium rotundiceps* Koch, det. C. Koch / Holotypus 1956, det. Kamp / Holotypus *Lathrobium rotundiceps* Koch / *Domene rotundiceps* (Koch) ♀, V.I. Gusarov det. 1993 / *Lobrathium rotundiceps* (Koch), det. V. Assing 2012” (NHMB).

Koch (1939) described *Lathrobium rotundiceps* Koch from a single female, from “Tienmuschan N.W. China”. Zheng (1988) included *Lobrathium rotundiceps* (Koch) in his key to species based on the original description. Recently, Assing (2012) redescribed the species based on the holotype, and pointed out that the type locality specified in the description (“Tienmuschan N.W. China”) was in “northeastern (not northwestern) China”.

**Figure 1.**
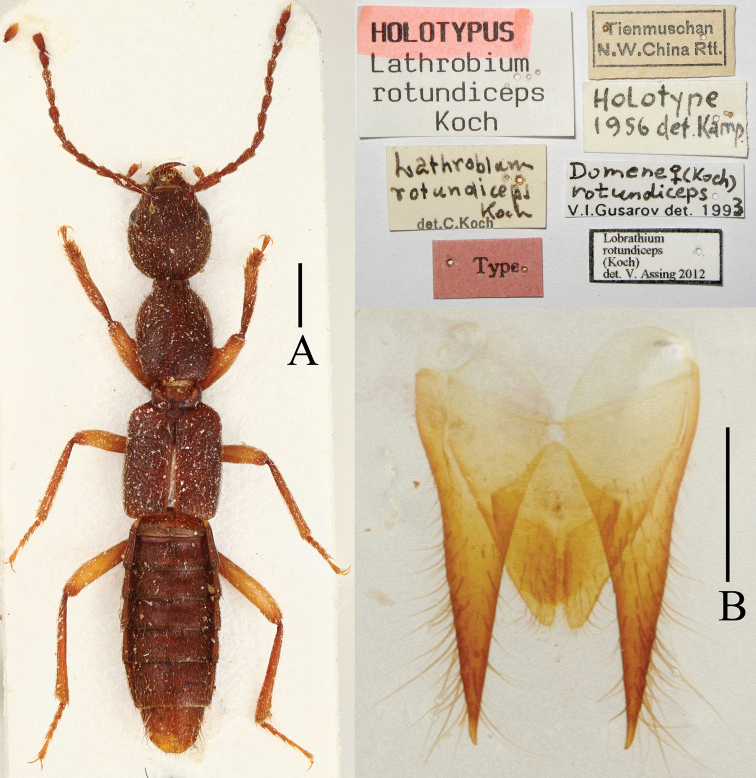
*Lobrathium rotundiceps*, holotype. **A** habitus **B** female tergites IX–X. Scales: **A** 1 mm, **B** 0.5 mm.

#### Additional material studied.

**China, Zhejiang** (12 ♂♂, 1 ♀): 3 ♂♂, Lin’an City, Tianmu Shan, 800–1150 m, 19–V–2006, Hu & Tang leg.; 5 ♂♂, Anji City, Longwang Shan, Qianmutian 4.8 km, 1050–1250 m, 08–VI–2012, Hu & Yin leg.; 4 ♂♂, Longwang Shan, Qianmutian, 1300 m, 29–V–2009, Yuan et al. leg.; 1 ♀, Longwang Shan, 1250–1450 m, 14–V–2013, Tang leg. (SNUC)

#### Description of male.

Body length: 8.87–9.62 mm, fore body length: 4.67–5.06 mm. HL/HW=1.08–1.10, PW/HW=0.87–0.96, EL/PL=0.92–1.01. Antenna 3.50–3.78 mm long.

**Figure 2.**
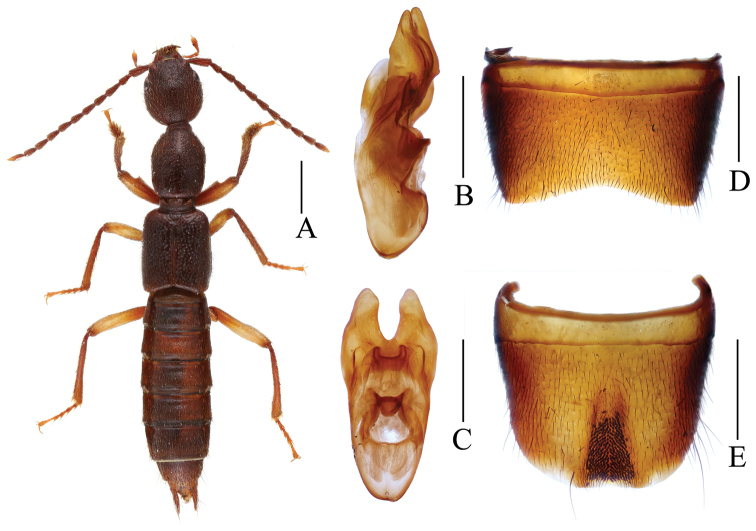
*Lobrathium rotundiceps*. **A** habitus **B** aedeagus in lateral view **C** aedeagus in ventral view **D** male sternite VII **E** male sternite VIII. Scales: **A** 1 mm, **B**–**E** 0.5 mm.

Sternite VII (Fig. 2D) strongly transverse and without impression, posterior margin broadly concave; sternite VIII (Fig. 2E) weakly transverse, with long and pronounced postero-median impression, this impression with numerous modified, stout and short black setae, posterior margin weakly concave in middle, near this concavity with cluster of dense fine setae; aedeagus (Figs 2B, 2C) 1.27–1.35 mm long, ventral process bifid in ventral view.

#### Comparative notes.

*Lobrathium rotundiceps* shares a bifid ventral process with *Lobrathium digitatum* Assing 2010 and *Lobrathium bidigitatum* Assing 2010 from Taiwan, but differs from them in many respects, particularly by much larger body size, the different shape and chaetotaxy of the male sternite VIII, and by the shape of the aedeagus.

#### Intraspecific variation.

The specimens from Qianmutian, 1050–1250 m are of darker coloration than those from Qianmutian, 1300 m, and Tianmu Shan, 800–1150 m.

#### Habitat and distribution.

The specimens were sifted from debris and moss in moist habitats. Tianmu Shan is a mountain with high biodiversity in Lin’an City in northwestern Zhejiang province in eastern China, its altitude ranging from 300 to 1506 m (Wu and Pan 2001). Longwang Shan is one peak of the Tiammu Shan range and situated about 5 km northwards of the West Tianmu Shan.

### Lobrathium luoxiaoense sp. n.

http://zoobank.org/8DE9CF08-3407-4FBB-814D-1CCF0AA0CC0A

http://species-id.net/wiki/Lobrathium_luoxiaoense

Fig. 3

#### Type material

(1 ♂, 1 ♀). **Holotype**, ♂: “China, Jiangxi, Pingxiang City, 27°34'15"N, 114°14'12"E, near Luxi County, Yangshimu Area, entrance, moss on rock in a stream, sifted, ca. 995 m, 16–VII–2012, Xiao-Bin Song leg. / Holotype ♂, *Lobrathium luoxiaoense*, sp. n., Li et al., det. 2013”. **Paratype**, ♀: “China, W. Jiangxi, Yichun City, Mingyueshan National Park, 27°35'43-41"N, 114°16'25"E, nr. Cableway station, moss on rock in a stream, sifted, ca. 1130 m, 13–VII–2013, Zi-Wei Yin leg.” (SNUC)

#### Description.

**Figure 3.**
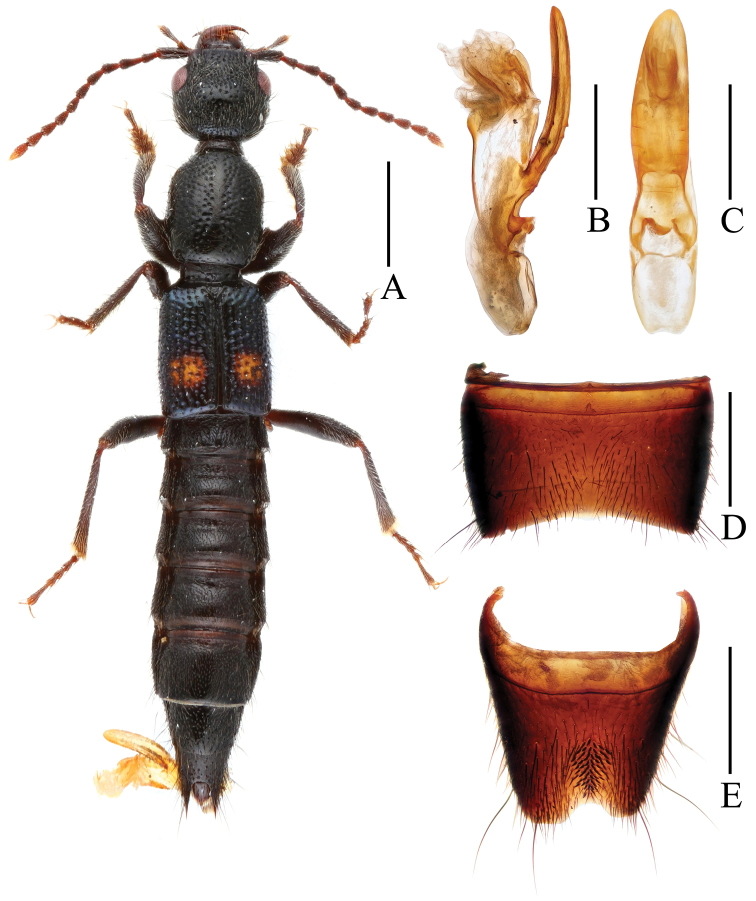
*Lobrathium luoxiaoense*. **A** habitus **B** aedeagus in lateral view **C** aedeagus in ventral view **D** male sternite VII **E** male sternite VIII. Scales: **A** 1 mm, **B**–**E** 0.5 mm.

Body length 7.56–7.67 mm, length of fore body 3.50–3.73 mm. Habitus as in Fig. 3A. Coloration: body black, elytra with blue hue, and anterior portion of posterior half with yellowish spot not reaching posterior and lateral margins; legs dark brownish with paler tarsi; antennae dark reddish.

Head distinctly transverse (HL/HW=0.90–0.91); posterior angles marked; punctation coarse and dense, sparser in median dorsal portion, interstices without microsculpture. Eyes large, more than half as long as distance from posterior margin of eye to neck. Antenna slender, 2.06–2.11 mm long.

Pronotum slender (PL/PW=1.17–1.21), 0.93–0.96 times as wide as head, lateral margins weakly convex in dorsal view; punctation dense, coarser than that of head, midline moderately broadly impunctate; interstices without microsculpture and glossy.

Elytra broad and moderately short (EL/EW=0.94–0.95, EW/PW=1.17–1.23, EL/PL=0.95–0.97); humeral angles marked; punctation coarse and arranged in distinct series, interstices without microsculpture and glossy. Hind wings fully developed.

Abdomen slightly broader than elytra; punctation fine and dense; posterior margin of tergite VII with palisade fringe; posterior margin of tergite VIII weakly convex, without appreciable sexual dimorphism.

**Male.** Sternite VII (Fig. 3D) with deep and very narrow and shallow median impression with pubescence, posterior margin broadly concave, weakly convex in middle; sternite VIII (Fig. 3E) weakly transverse, with deep and pronounced postero-median impression, this impression with numerous (about 60) modified, stout and short black setae, posterior excision relatively small, near posterior excision with long dark setae; aedeagus (Figs 3B, C) 1.42 mm long, ventral process long and broad, apically convex in ventral view.

**Female.** Posterior margin of tergite VIII weakly convex in middle; posteriorly margin of sternite VIII broadly convex.

#### Etymology.

The specific epithet (adjective) is derived from the Luoxiao Shan range where the type locality is situated.

#### Comparative notes.

This species is highly similar to *Lobrathium anatitum* Li & Li (2013) in external (habitus, position of the elytral spots) and male sexual characters (modifications of the male sternites VII and VIII; shape of the ventral process of the aedeagus). The new species differs from *Lobrathium anatitum* by the narrower median impression of the male sternite VII, by the less extensive median cluster of modified setae and the smaller posterior excision of the male sternite VIII, as well as by the shape of the ventral process of the aedeagus (apex more acute in ventral view). For illustrations of *Lobrathium anatitum* see Li et al. (2013).

#### Habitat and distribution.

The specimens were sifted from moss on stones in two streams, Jiangxi, East China.

## Supplementary Material

XML Treatment for Lobrathium rotundiceps

XML Treatment for Lobrathium luoxiaoense
